# Involvement of the Cannabinoid CB1 Receptor in Modulation of Dopamine Output in the Prefrontal Cortex Associated with Food Restriction in Rats

**DOI:** 10.1371/journal.pone.0092224

**Published:** 2014-03-14

**Authors:** Laura Dazzi, Giuseppe Talani, Francesca Biggio, Cinzia Utzeri, Valeria Lallai, Valentina Licheri, Stefano Lutzu, Maria Cristina Mostallino, Pietro Paolo Secci, Giovanni Biggio, Enrico Sanna

**Affiliations:** 1 Department of Life and Environmental Sciences, Section of Neuroscience and Anthropology, Centre of Excellence for the Neurobiology of Dependence, University of Cagliari, Monserrato, Cagliari, Italy; 2 Institute of Neuroscience, National Research Council, Monserrato, Cagliari, Italy; Duke, Psychology and Neuroscience, United States of America

## Abstract

Increase in dopamine output on corticolimbic structures, such as medial prefrontal cortex (mPFC) and nucleus accumbens, has been related to reward effects associated with palatable food or food presentation after a fasting period. The endocannabinoid system regulates feeding behavior through a modulatory action on different neurotransmitter systems, including the dopaminergic system. To elucidate the involvement of type 1 cannabinoid receptors in the regulation of dopamine output in the mPFC associated with feeding in hungry rats, we restricted the food availability to a 2-h period daily for 3 weeks. In food-restricted rats the extracellular dopamine concentration in the mPFC increased starting 80 min before food presentation and returned to baseline after food removal. These changes were attenuated in animals treated with the CB1 receptor antagonist SR141716. To better understand how food restriction can change the response of mesocortical dopaminergic neurons, we studied several components of the neuronal circuit that regulates dopamine output in the mPFC. Patch-clamp experiments revealed that the inhibitory effect of the CB1 receptor agonist WIN 55,212-2 on GABAergic sIPSC frequency was diminished in mPFC neurons of FR compared to fed ad libitum rats. The basal sIPSC frequency resulted reduced in mPFC neurons of food-restricted rats, suggestive of an altered regulation of presynaptic GABA release; these changes were accompanied by an enhanced excitability of mPFC and ventral tegmental area neurons. Finally, type 1 cannabinoid receptor expression in the mPFC was reduced in food-restricted rats. Together, our data support an involvement of the endocannabinoid system in regulation of dopamine release in the mPFC through changes in GABA inhibitory synapses and suggest that the emphasized feeding-associated increase in dopamine output in the mPFC of food-restricted rats might be correlated with an altered expression and function of type 1 cannabinoid receptor in this brain region.

## Introduction

The rewarding effects associated with reinforcers such as palatable food or food presentation after starvation have been related with an increased dopamine output in corticolimbic structures including the medial prefrontal cortex (mPFC) and the nucleus accumbens (NAcc) [1]–[3]. Lesions of mesencephalic dopamine neurons by 6-hydroxydopamine result in a severe hypophagic syndrome [4], whereas an increase in the amount of dopamine, induced by dopamine reuptake inhibitors, has been found to promote food intake [5]. The marked increase in dopamine output in the mPFC induced by exposure to palatable food [6], together with the high levels of endocannabinoids (eCB) detected in limbic areas of food-restricted (FR) animals [7], suggest that the eCB system might contribute to the regulation of the activity of dopaminergic neurons that project to the mPFC.

It is well known that the eCB system contributes to the psychotropic and hunger-stimulating effects of cannabis [8]–[10]. Both exogenous (Δ9-tetrahydrocannabinol) and endogenous (anandamide, AEA; 2-Arachidonoylglycerol, 2AG) cannabinoid receptor agonists thus induce a state of overeating in rats [11]–[12]. Indeed, changes in eating motivation associated with cannabis use might reflect an important role for the eCB system in the control of appetite, feeding behavior, energy metabolism, and body weight [12].

Consistent with such role for eCBs, administration of the cannabinoid receptor antagonist SR141716 was found to attenuate the increase in the extracellular concentration of dopamine in the rat NAcc induced by exposure to a novel highly palatable food [13], whereas acute administration of Δ9-tetrahydrocannabinol recapitulates the effect of intraoral sucrose administration on *in vivo* dopamine transmission in the same brain area [14].

Brain regions with a moderate to high density of the type 1 cannabinoid (CB1) receptor protein and abundant of CB1 receptor mRNA are implicated in the control of feeding and body weight [15]–[17]. Moreover, changes in CB1 receptor expression or eCB signaling are associated with metabolic impairment or diet-induced obesity [9], [18]–[19].

In light of this evidence and given the important role of CB1 receptors in the mesocortical circuitry directed in the control of dopamine output [20]–[22], we have here investigated the potential involvement of the eCB system in the regulation of dopamine output in the mPFC during food anticipation and consumption in rats exposed to food restriction (FR) diet. In particular, we examined the effects of CB1 receptor ligands on dopamine output in the mPFC and the NAcc shell, two brain areas implicated in the response to appetitive stimuli [6], [14], with the use of microdialysis technique in freely moving FR rats. We also evaluated CB1 receptor function in FR rats by whole-cell patch-clamp recording of γ-aminobutyric acid type A (GABAA) receptor–mediated spontaneous inhibitory postsynaptic currents (sIPSCs) in brain slices containing mPFC pyramidal neurons. Current-clamp analysis was also performed in order to study the effect of food anticipation and consumption on excitability of mPFC glutamatergic pyramidal neurons and ventral tegmental area (VTA) principal dopaminergic cells in FR rats. Finally, we examined whether FR affected CB1 receptor abundance in the mPFC by Western blot and confocal microscopy analysis.

## Experimental Procedures

### Animals

Male Sprague Dawley CD young-adult rats (Charles River, Como, Italy) were bred in our animal facility and maintained under an artificial 12-h-light, 12-h-dark cycle (lights on from 08∶00 to 20∶00 hours), a constant temperature of 22° ±2°C, and a relative humidity of 65%. They had free access to water and standard laboratory food at all times until the food restriction regimen was applied. All efforts were made to minimize animal suffering. Animal care and handling throughout the whole experimental procedures were made in accordance with the European Communities Council Directive of 24 November 1986 (86/609/EEC). The experimental protocols were also approved by the Animal Ethics Committee of the University of Cagliari.

### Food Restriction Paradigm

Rats with a body mass of 200 to 230 g were assigned either to a control group that received food and water ad libitum or to the FR group. As described previously [23], FR animals were allowed to access to their daily meal (rat food pellets; Standard Diet GLP, Mucedola, Italy) for only 2 h, from 11∶00 to 13∶00 hours, whereas water was available ad libitum. Body weight and food consumption were measured daily during the 3-week period in which the FR regimen was applied. The amount of food consumed by FR animals during the 2 h in which the food was available, increased gradually to achieve a constant level (21.7±0.5 g/2 h) within 10 to 14 days, being slightly but significantly (*P*<0.05) lower than what observed in rats fed ad libitum during 24 h (24.6±0.3 g/24 h).

The amount of food eaten by FR as well as rats fed ad libitum did not significantly change after neither acute administration of WIN 55,212-2 (22.2±0.8 g/2 h) nor SR141716 (20.7±0.7 g/2 h).

### Microdialysis and Drug Treatment

**Figure 1:**
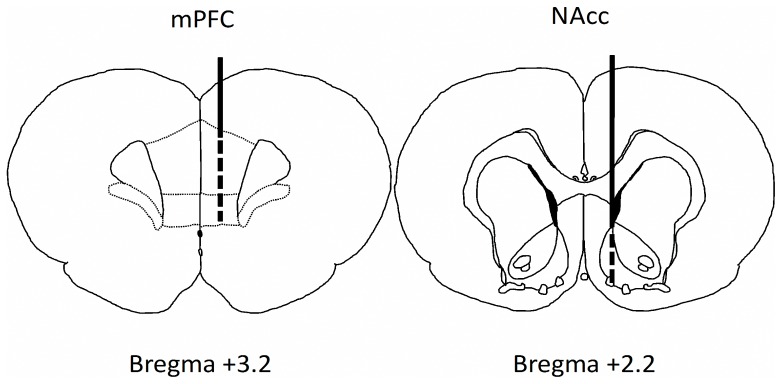
Schematic representation of the localization of the dialysis probe in the mPFC and NAcc shell.

Rats were anesthetized by intraperitoneal (i.p.) injection of chloral hydrate (0.4 g/kg), and a concentric dialysis probe was inserted at the level of the mPFC (A +3.2, ML +0.8, V –5.3 relative to the bregma) or NAcc shell (A +2.2, ML +1, V −7.8), as shown in Fig. 1, according to the Paxinos atlas [24]. The active length of the dialysis membrane (Hospal Dasco, Bologna, Italy) was restricted to 4 mm for mPFC and 2 mm for NAcc. In the mPFC the lenght of the dialitycal membrane allowed to sample from both infralimbic and prelimbic cortices. Experiments were performed in freely moving rats ∼24 h after probe implantation to allow recovery from surgery procedures. Ringer’s solution [3 mM KCl, 125 mM NaCl, 1.3 mM CaCl2, 1 mM MgCl2, 23 mM NaHCO3, 1.5 mM potassium phosphate (pH 7.3)] was pumped through the dialysis probe at a constant rate of 2 µl/min. Samples of dialysate were collected every 20 min from 8∶30 to 15∶00 hours and immediately analyzed for dopamine by HPLC with electrochemical detection, as previously described [25]; the detection limit for dopamine was 2 fmol per injection. The average neurotransmitter concentration in the first two samples was taken as 100%, and all subsequent values were expressed as means ± SEM relative to the basal value. The mean in vitro recovery of the probes was 15±3%. All probes were tested before implantation, and those with a recovery value outside of this range were not used. The absolute concentration of dopamine was not corrected for this value. At the end of each experiment, the placement of the probe was verified histologically. All rats in which the probe was located outside of the target region were excluded from the analysis. Rats were subjected to acute or long-term (twice a day for 21 days) treatment with SR141716 (1 mg/kg, i.p.) dissolved in ethanol/Tween 80/saline (1∶1: 16, v/v/v). In a separate group of FR rats, SR141716 was administered locally in the mPFC through the dialysis probe via inverse dialysis. In this case a 1 mM solution of SR141716 was prepared in ethanol/Tween 80/Ringer (1∶1: 16, v/v/v) then diluted to 10 µM to be perfused. Forty min before food presentation basal Ringer solution was replaced with the solution containing SR141716. As the probe recovery was 15±3%, we assume that the amount of SR141716 reaching the brain was approximately 1.2–1.7 µM. WIN 55,212-2 was dissolved in distilled water containing one drop of Tween 80 per milliliter and administered acutely at a dose of 5 mg/kg (i.p.). WIN 55,212-2 and SR141716 were administered in a volume of 3 ml/kg, and an identical volume of vehicle was administered as a control.

### Recording of Spontaneous Exploratory Behavior

Spontaneous exploratory behavior was measured by a motility meter (Omnitech Electronics Inc.) in FR rats at different time points following the three-week period of FR regimen: during the anticipatory phase (1 h and 5 min before food presentation), at the end of feeding and 2 h after food removal. A group of rats fed ad libitum were tested as controls. Eight rats were used for each group. On the test day, rats were brought into the test room under standardized environmental conditions and acclimated for 1 h before the beginning of the experiment. They were then individually placed in the test cage. Each cage consisted of a 41×41×30 cm specially designed sound-attenuating shells made of polypropylene and an expanded PVC sheet equipped with two sets of photocells located at the bottom of both cage sides, projecting horizontal infrared beams. Animals were isolated from noise of the recorder and printer used to acquire the data by placing these devices in a different room. Horizontal activity (number of horizontal infrared beams interruptions), total distance traveled (centimeters), locomotion time (seconds), and rest time (seconds) were acquired with a recorder (Omnitech Electronics, Inc.). Recordings were performed every 5 min, starting immediately after placing the rat in the cage, over a period of 30 min.

### Preparation of mPFC and VTA Slices and Whole-cell Patch-clamp Recordings

Coronal and horizontal slices containing the mPFC and the VTA, respectively, were prepared from rats fed ad libitum and FR animals at different time points relative to the 2-h feeding period (60 and 5 min before food presentation; at the end of the feeding period; and 1 h after food removal) as previously described for the hippocampus [26]–[27].

Whole-cell patch-clamp recordings from mPFC neurons were performed as previously described [26]–[27]. In brief, spontaneous GABA type A (GABAA) receptor–mediated ISPCs (sIPSCs) were recorded in the presence of the ionotropic glutamate receptor antagonist kynurenic acid (1 mM) with the use of an Axopatch 200-B amplifier (Axon Instruments, Sunnyvale, CA, USA), and they were filtered at 2 kHz and digitized at 5 kHz. Off-line analysis of sIPSCs was performed with Minianalysis 6.0 software (Synaptosoft, Decatur, GA, USA). For application of the depolarization-induced suppression of inhibition (DSI) protocol, we recorded sIPSCs under control conditions for 3 min and then imposed a depolarization step from –65 to 0 mV on the postsynaptic neuron for 5 s. DSI was calculated from the sIPSC amplitudes and frequencies measured before (baseline) and after the depolarization step. In experiments evaluating the effect of various drugs perfusion, the last 180 s of each recording were used for statistical comparison with baseline. In a different set of experiments, neuronal excitability was studied in the current-clamp mode by evaluating spontaneous and evoked action potential (AP) firing rate in VTA and mPFC neurons, respectively. Dopamine neurons in the VTA were identified by the presence of an I_h_ current, as previously reported [35], which was evoked by incrementally hyperpolarizing (steps of 10 mV) the membrane from an holding potential of −70 mV. The resting potential in spontaneously firing dopamine neurons was measured by single cell patch-clamp recordings in the current-clamp mode.

### Immunoblot Analysis

At the end of the food restriction protocol, rats were killed by decapitation for immunoblot analysis. The brain was rapidly removed, and the mPFC was dissected on ice, weighed, and stored at –80°C until analysis. Total protein was extracted from the tissue with the use of a kit (Membrane and Cytoplasmatic Protein Extraction kit, Bio Basic Inc, Markham Ontario, Canada), and the protein concentration of the extract was determined with the use of a DC Protein Assay kit (Bio-Rad, Milan, Italy). The extract (40 µg of protein in 15 µl) was incubated for 10 min at 70°C and then fractionated by SDS-polyacrylamide gel electrophoresis (NuPAGE Novex 4–12% Bis-Tris Midi Gel, Life Technologies, Monza, Italy). The separated proteins were transferred to a polyvinylidene difluoride membrane (Immobilon-P; Millipore, Milan, Italy) with the use of a Criterion Blotter (Bio-Rad), after which the membrane was exposed to 5% nonfat dried milk in Tris-buffered saline containing 1% Tween 20 (TBS-T) before an over-night incubation for at 4°C with rabbit polyclonal antibodies to CB1 receptors (1∶500) (Calbiochem, Merk-Millipore, Milan, Italy) and mouse monoclonal antibodies to glyceraldehyde-3-phosphate dehydrogenase (GAPDH, 1∶5000) (Millipore) diluted in TBS-T containing 5% nonfat dried milk. The membrane was then incubated for 1 h at room temperature with horseradish peroxidase–conjugated goat secondary antibodies (anti-rabbit, Sigma, Milan, Italy; anti-mouse, Millipore, Milan, Italy) in TBS containing 5% nonfat dried milk and 0.5% Tween 20, after which immune complexes were detected with Luminata Crescendo and Forte substrates (Millipore). The intensity of immunoreactive bands was quantitated with the use of a Geliance 600 Chemi Imaging System (Perkin Elmer, Monza, Italy) and GeneTools software (Perkin Elmer). The intensity of CB1 receptor bands was normalized by that of the corresponding GAPDH band.

### Antibodies

The following primary antibodies were used in this study: rabbit anti-CB1 (1∶500; Millipore, Billerica, MA), rabbit anti-CCK (1∶500; Millipore, Billerica, MA), mouse anti-parvalbumin (1∶2000; Millipore, Billerica, MA), guinea pig anti-GAD65 (1∶1500; Synaptic System, Goettingen Germany).

### Tissue Preparation and Immunofluorescence

Rats were deeply anesthetized with Equithesin (1 g sodium pentobarbital, 4.251 g choral hydrate, 2.125 g MgSO4, 12 ml EtOH, 43.6 ml propylene glycol, adjusted to a total volume of 100 ml with distilled water) at 0.3 ml/g and transcardially perfused with 4% paraformaldehyde in 0.1 M phosphate buffer, pH 7.3, at 1 h and 5 min before food presentation, during eating phase and 1 h after food removal. Brains were then quickly removed and post-fixed for 4 h with 4% paraformaldehyde. After thorough rinsing, brains were cryoprotected in 20% sucrose overnight at 4°C. The day after sagittal sections of 50 µm thickness were obtained with a vibratome (Leica Microsystems, Milan, Italy) and maintained in antifreeze solution (30% ethylene glycol, 20% glycerol and 50% 0.05 M phosphate buffer v/v) at −20°C. Indirect immunohistochemistry was performed with free-floating sections as follows: (Method 1, double labeling): the sections were washed with PBS, permeabilized for 1 h with 0.1% Triton X-100 in PBS (PBS-T), incubated for 1 h with 10% normal donkey serum (Jackson ImmunoResearch, West Grove, PA, USA) in PBS-T and incubated overnight with CCK primary antibody. The sections were then rinsed in PBS-T and incubated with CY3-conjugated polyclonal donkey anti-rabbit antibody for 2 h at RT (1∶1500 in PBS-T; Millipore). The sections were washed several time in PBS-T, re-incubated for 1 h with 10% of goat serum in PBS-T and incubated overnight at 4°C with CB1 receptor primary antibody.

The day after the sections were washed several times and incubated for 1 h with CY2-conjugated polyclonal goat anti-rabbit antibody for 2 h a RT (1∶750 in PBS-T; Millipore). After several washing in PBS-T, nuclei were counterstained with 1 µg/ml Hoechst 33342 in PBS for 30 min, rinsed again in PBS and coverslipped with gelatin-glicerol mounting medium (Sigma-Aldrich, Milan, Italy). (Method 2, triple labeling): serial sections were used and processed as described for method 1. After permeabilization with 0.1% Triton X-100 and incubation with normal donkey serum, the sections were incubated with a combinations of three primary antibodies (anti CB1 receptor, anti-parvalbumin and anti GAD 65) raised in different species. The sections were then rinsed in PBS-T, incubated with the appropriate secondary antibodies, raise either in goat or donkey, conjugated to one of the following fluorophores cyanine-derived Cy2 (1∶750 in PBS-T; Millipore), Cy5 (1∶1500 in PBS-T; Millipore) and Dylight 594 (1∶1500 in PBS-T; Jackson Immunoreasearch, West Grove, PA), incubated with Hoescht 33342 and coverslipped with gelatin glycerol mounting medium (Sigma-Aldrich, Milan, Italy). Control sections not exposed to primary antibodies did not yield positive staining (results not shown).

### Confocal Microscopy and Data Analysis

Acquisitions were carried out using a Leica TCS SP5X Inverted Supercontinuum Confocal Laser Scanning microscope (Leica Microsystems, Heidelberg, Germany) equipped with a diode 405 laser and a white laser and using the multichannel acquisition mode to avoid fluorescence crosstalk. Images (2048×2048 pixels) were obtained with a Plan Apo 63X oil immersion objective NA 1.4. For quantification, confocal settings were chosen to ensure that the signal obtained from co-localization of CB1/CCK and CB1/PV/GAD35 were fully resolved within the dynamic range of detection (8 bit, 0–255) and no less or saturation of signal did occur. Acquisitions were performed in the section III and IV of the prefrontal cortex for co-localization study of CB1/PV/GAD65 and CB1/CCK, respectively. Same setting was used for all images captured consisting of identical detector gain, amplifier gain, amplifier offset, pinhole diameter (1 Airy unit), laser power excitation, scan mode and speed, frame size (2048×2048 pixels, 8 bit) and magnification of 1.4×10^−2^ µm^2^/pixel. In our experiments for excitation of the Hoescht 33342, the 405 nm wavelength was used and the emission was detected in the 417–486 nm range; for excitation of the Cy2, the 492 nm wavelength was used and the emission was detected in the 497–548 nm range; for the Cy3 the 550 nm wavelength and the 660–1250 nm range were used; for the Cy5 the 650 nm wavelength and the 655–730 nm range were used; and for the DyLight 594 the 594 nm wavelength and the 604–650 nm range were used respectively for excitation and emission. The images were processed with the image-analysis program images NIH Image J software (http://rsb.info.nih.gov/nih-image). Control images were obtained using sections incubated with secondary antibody alone and/or without antibodies.

To determine the density of CB1 receptor and their co-localization with CCK or GAD65 proteins, images were first segmented using a threshold that maximized the selection of immunofluorescent puncta over background labeling and then processed with the “co-localization” module, in which a mask is generated from the comparison of two different confocal channels. The number and density of puncta were then calculated with ImageJ (version 1.46a). It should be noted that the ratio between the intensity of CB1 receptor immunofluorescent puncta and diffuse extrasynaptic labeling is considerably lower in young animals compared to adult, making it impossible to select an intensity threshold that allows detection of all clusters without also including some background fluorescence. Because of this the number of immunopositive puncta will be either overestimated, due to the inclusion of background labeling, or underestimated, by elimination of the less intensely fluorescent clusters. This bias can be corrected in part by selecting clusters according to an appropriate size threshold during the automated counting procedure. In the present analysis we found that selecting clusters larger than 0.084 µm^2^ (corresponding to five pixels) resulted in counts that approximated the number of clusters determined by Leica software. Another important issue concerns the analysis of co-localization. Because the overlap between individual CCK/CB1 and CB1/GAD65 receptor positive clusters is only partial, the resulting co-localized clusters (i.e., pixels expressing both fluorescence channel signals) will have a considerably smaller size. To take this into account, we set a smaller size threshold (250 nm, corresponding to two pixels) for co-localized clusters. The co-apposition of CB1 receptor clusters with CCK or GAD-65 positive boutons was estimated visually in the three orthogonal planes. For presentation, digital micrographs were processed with software Leica and only minimal contrast adjustments were made. Files were then imported into Adobe Photoshop (Adobe Systems, San Jose, CA), where images were cropped.

### Statistical Analysis

Data are presented as means ± SEM. Electrophysiological and immunoblot results were compared by one-way or two-way analysis of variance (ANOVA) or Student’s t Test. Microdialysis data were compared among groups with two-way ANOVA for repeated measures, with factors of treatment and time points. The raw values of dopamine concentration were used for the analysis, with absolute basal concentrations given in Results. Normal distribution of data was verified by Skewness and Kurtosis evaluation with Graph Pad Prism 5.0. Post hoc comparisons were performed with Newman-Keuls or Bonferroni’s test where reported. A *P* value of <0.05 was considered statistically significant for all experiments.

## Results

### Effect of Food Anticipation and Consumption on Extracellular Dopamine Concentration in the mPFC and its Modulation by CB1 Receptor Ligands in FR Rats

**Figure 2:**
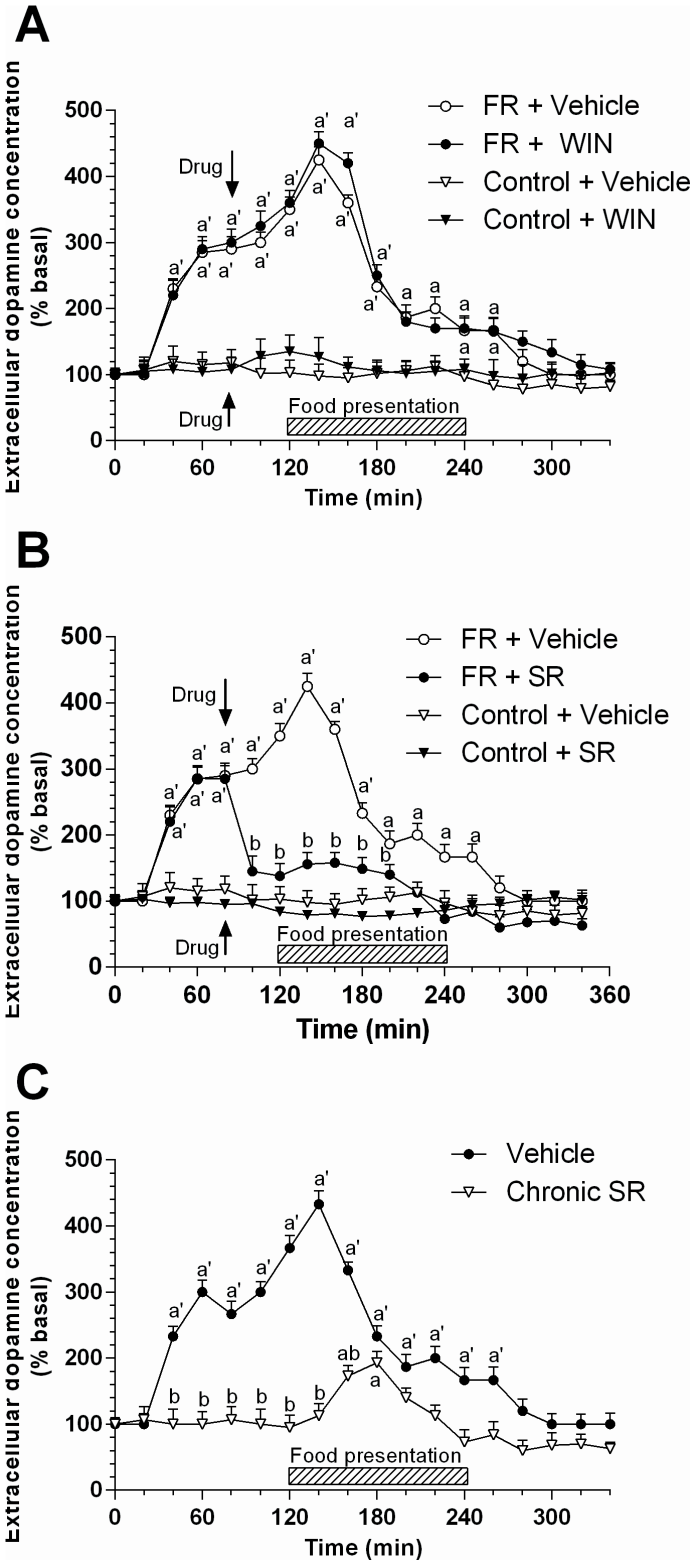
Changes in extracellular dopamine concentration in the mPFC during anticipation and consumption of food in FR rats: modulation by CB1 receptor ligands. Rats were trained to consume their daily meal within a 2-h period (11∶00 to 13∶00 hours) (circle symbols), or fed ad libitum (triangle down symbols), and microdialysis samples were collected from the mPFC before, during, and after food presentation. Animals also received an acute administration of vehicle (open symbols, n = 5), WIN 55212-2 (5 mg/kg, i.p., closed symbols, n = 5) (A), or SR141716 (1 mg/kg, i.p., closed symbols, n = 4) (B) at 40 min before food presentation or were chronically treated with vehicle (open symbols, n = 5) or SR141716 (1 mg/kg, i.p., closed symbols, n = 5) twice a day for 21 days (C). Data are expressed as a percentage of basal values and are means ± SEM. ^a^ *P*<0.05, ^a’^ *P*<0.01 versus basal values; ^b^ *P*<0.01 versus corresponding vehicle value.

We started our study by assessing the basal extracellular concentration of dopamine in the mPFC of rats trained to consume their daily meal within a 2-h period (11∶00 to 13∶00 hours) which was not significantly different from that of rats fed ad libitum (12.58±2.95 fmol per 40-µl sample for FR rats vs 13.54±3.05 fmol per 40-µl sample for rats fed ad libitum). In FR rats the extracellular dopamine concentration was significantly increased as early as 80 min before food presentation and increased further to +200% of basal values by 20 min before feeding (Fig. 2A). It increased even further during food intake to reach a maximal value of +320% at 20 min after food presentation. It then declined to a value of +100% at the end of the feeding period before returning to basal values at 40 min after food removal. In rats, who had free access to food during all day, the extracellular concentration of dopamine did not change by more than 20% during the experiment (Fig. 2A). To evaluate the possible role of CB1 receptors in such marked increase of extracellular dopamine concentration in FR rats, we examined the effect of acute stimulation or blockade of the receptor with WIN 55,212-2 (5 mg/kg, i.p., Fig. 2A) or SR141716 (1 mg/kg, i.p., Fig. 2B), respectively. The doses used for our experiments are in the range that has previously been described for microdialysis experiments [28]–[29], [22]. Each drug was administered to a different group of animals 40 min before food presentation. Whereas stimulation of CB1 receptors with WIN 55212-2 did not further increase dopamine output induced by food presentation, acute administration of SR141716 completely antagonized this response (Fig. 2B) with an effect starting immediately following drug injection. In rats that were not subjected to food restriction the same doses of SR141617 or WIN 55,212-2 induced a small decrease (−28%) and no effect, respectively, on dopamine output (Fig. 2A and B). To evaluate whether this action of SR141716 was elicited by blockade of CB1 receptors located in the mPFC, we studied the effect of the local infusion of this drug through the dialysis probe on the increase in dopamine output induced by food presentation in FR rats. Local administration of SR141716 elicited an effect that wasn’t significantly different from that induced by intraperitoneal administration of the drug (data not shown). For data presented in Fig. 2A, ANOVA revealed a significant main effect of food presentation [*F*(3, 360) = 3.2564, *P*<0.01], a non significant effect of WIN 55,212-2 administration [*F*(18, 360) = 0.2486, *P* = 0.1248], and a non significant interaction between factors [*F*(54, 360) = 0.8596, *P* = 0.9536]. For data showed in Fig. 2B, ANOVA revealed a significant main effect of treatment [*F*(3, 324) = 12.0308, *P*<0.01], a significant main effect of repeated measures [*F*(18, 324) = 2.1568, *P*<0.01], and a significant interaction between factors [*F*(54, 324) = 1.2687, *P*<0.01]. For the effect of local administration of SR141716, ANOVA revealed a significant main effect of treatment [*F*(3, 360) = 11.3596, *P*<0.01], a significant main effect of repeated measures [*F*(18,360) = 1.9587, *P*<0.01], and a significant interaction between factors [*F*(54, 360) = 1.0526, *P*<0.01].

### Chronic Administration of SR141716 Prevents the Increase in Dopamine Output in the mPFC Induced by Food Anticipation and Consumption in FR Rats

It has been shown that after chronic administration, tolerance develops to some of the behavioral and biochemical effects of SR141716 [30] but not to its anorexic effect [31]. To evaluate whether tolerance would develop also to the ability of this drug to antagonize the increase in dopamine output in the mPFC induced by food restriction, we evaluated the effect of chronic administration of SR141716 (1 mg/kg, i.p., twice a day for 21 days) in FR rats. The basal extracellular concentration of dopamine was not significantly affected by such long-term treatment (11.87±2.68 fmol per 40 µl sample; *P* = 0.175 vs rats fed ad libitum). In animals chronically treated with SR141716, the increase in dopamine output during the anticipatory phase was abolished, whereas that during the consummatory phase was markedly reduced from a maximum of +350% of basal to +80% and delayed (Fig. 2C). ANOVA revealed a significant main effect of treatment [*F*(1, 189) = 1.947, *P*<0.001], a significant main effect of repeated measures [*F*(18, 189) = 0.763, *P*<0.001], and a significant interaction between factors [*F*(18, 189) = 4.951, *P*<0.001].

### Effect of Food Anticipation and Consumption on Extracellular Dopamine Concentration in the NAcc and its Modulation by CB1 Receptor Ligands in FR Rats

**Figure 3:**
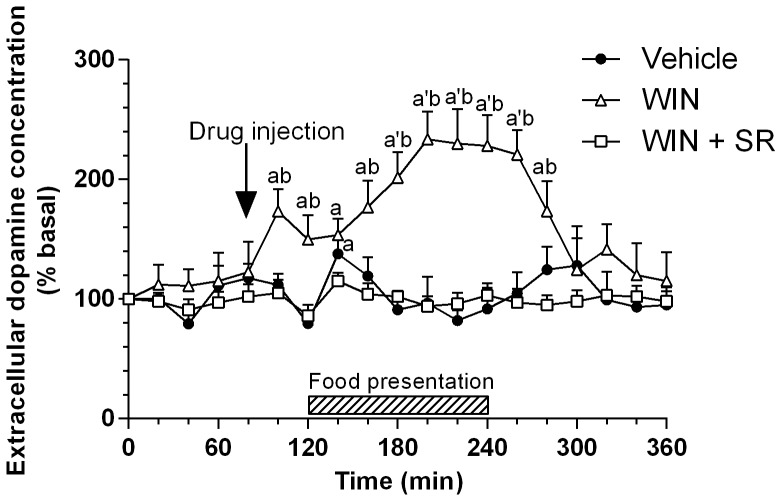
Changes in extracellular dopamine concentration in NAcc shell during anticipation and consumption of food in FR rats: modulation by CB1 receptor ligands. Rats were trained to consume their daily meal during a 2-h period, and microdialysis samples were collected from the NAcc shell before, during, and after food presentation. Animals received an acute administration of vehicle (closed cycles; n = 5), WIN 55,212-2 (5 mg/kg, i.p., open triangles; n = 4), or a combination of WIN 55, 212-2 (5 mg/kg, i.p.) and SR141716 (1 mg/kg, i.p.)(open squares; n = 6) at 40 min before food presentation. Data are expressed as a percentage of basal values and are means ± SEM. ^a^ *P*<0.05, ^a’^ *P*<0.01 versus basal values; ^b^ *P*<0.01 versus corresponding vehicle value.

Consistent with previous observations [14], mesolimbic dopaminergic neurons projecting to the NAcc shell did not respond significantly to anticipation of a familiar food, and food presentation induced only a small and transient increase (to +40% of basal values) in dopamine output in this brain area of FR rats (Fig. 3). In contrast, FR rats subjected to acute administration of WIN 55,212-2 (5 mg/kg, i.p.), at 40 min before food presentation and at a dose which per se induced a small (+28%) and transient (40 min) increase in dopamine output in rats fed ad libitum (data not shown), showed a significant increase in the extracellular concentration of dopamine during both the anticipatory (up to +60% of basal) and consummatory (+130%) phases (Fig. 3). Moreover, as expected [13], the concomitant administration of SR141716, which per se did not significantly modify basal extracellular dopamine concentration, completely antagonized the effect of WIN55,212-2 on this parameter.

ANOVA revealed a significant main effect of treatment [*F*(2,284 ) = 123.20, *P*<0.01], a significant main effect of repeated measures [*F*(18, 284) = 164.51, *P*<0.01], and a significant interaction between factors [*F*(36, 284) = 182.27, *P*<0.01].

### Changes in Exploratory Behavior in Rats Exposed to Food Restriction

**Figure 4:**
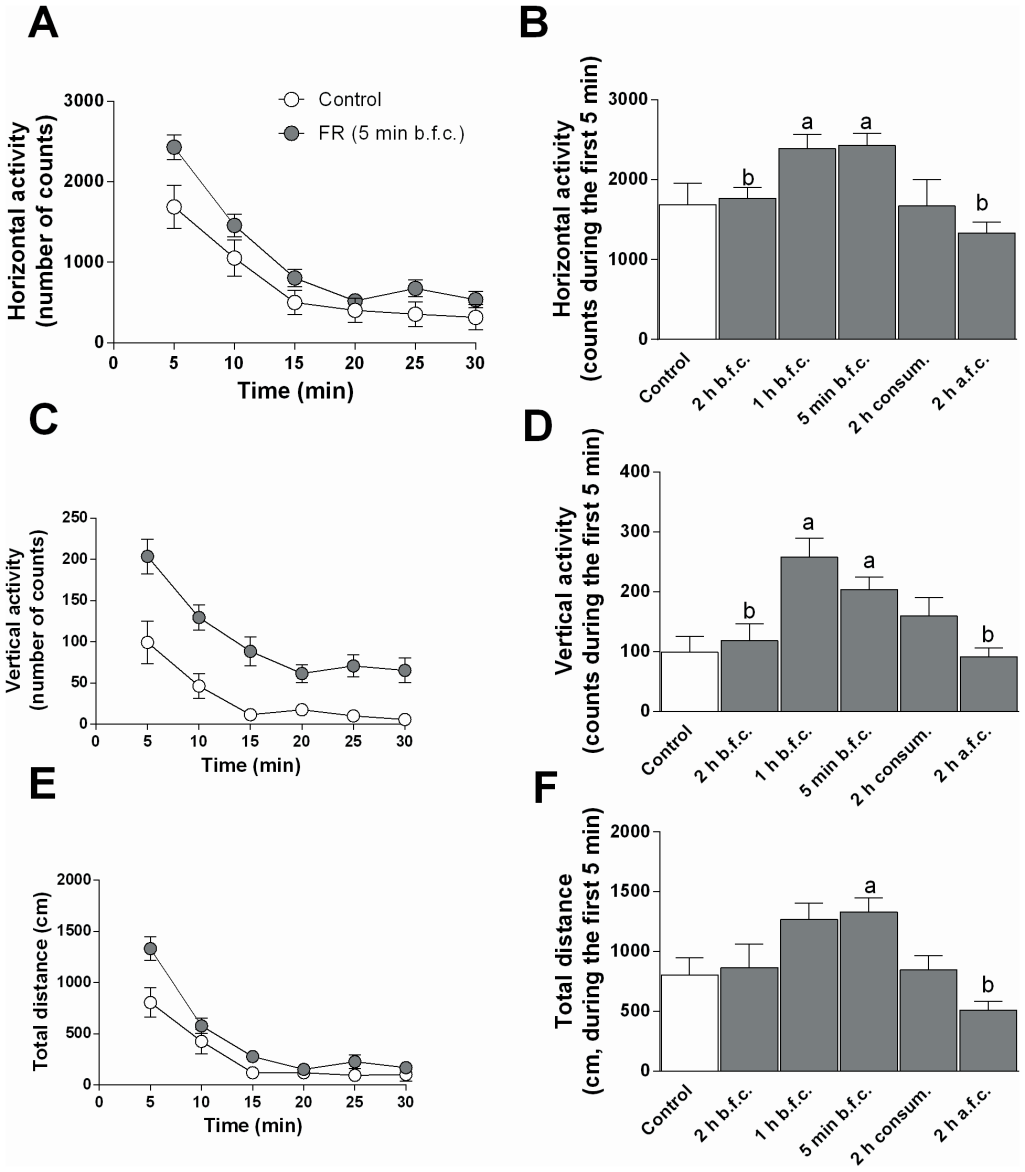
Spontaneous exploratory activity in FR and animals fed ad libitum. Exploratory activity was assessed in a motility meter after 3 weeks of FR. The different parameters measured were: horizontal activity (**A, B**), vertical activity (**C, D**), and total distance travelled (**E, F**) that were averaged in bins of 5 min, for 30 min (scatter plots) or during the first 5 min only (bar graphs). b.f.c., before food consumption; a.f.c, after food consumption; consum., consummatory phase. Data in graphs are means ± SEM (n = 4 per group) of the absolute values of the different measures. ^a^ *P*<0.05 versus animals fed ad libitum, ^b^ *P*<0.05 vs 5 min before food consumption group, one-way ANOVA, post hoc Newman-Keuls multiple comparison test.

Because previous studies have shown that food restriction increases arousal in many mammalian species [32], we wanted to test whether in our FR animals, anticipation and consumption of food were associated with an altered spontaneous exploratory behavior assessed in a motility meter. The data illustrated in Fig. 4 indicate that FR rats tested 5 min before food consumption displayed an increased horizontal and vertical activity, and traveled a greater distance, compared to animals fed ad libitum, as assessed during the initial 5 min of testing (Fig. 4A–F; one-way ANOVA; horizontal activity, F(5,49) = 5.267, ^a^ *P*<0.05 vs rats fed ad libitum; vertical activity, F(5,49) = 6.232, ^a^ *P*<0.05 vs rats fed ad libitum; total distance, F(5,49) = 5.640, ^a^ *P*<0.05 vs rats fed ad libitum ). Moreover, FR animals tested 2 h after or 2 h before food consumption, when dopamine levels are similar to those observed in animals fed ad libitum, showed a significant decrease in exploratory activity compared to FR rats tested 5 min before food consumption (Fig. 4A–F; ^b^ *P*<0.05 vs FR 5 min before, post-hoc analysis with Newman-Keuls)).

### Changes in CB1 Receptor-mediated Regulation of GABAergic Synapses in the mPFC of Rats Exposed to Food Restriction

**Figure 5:**
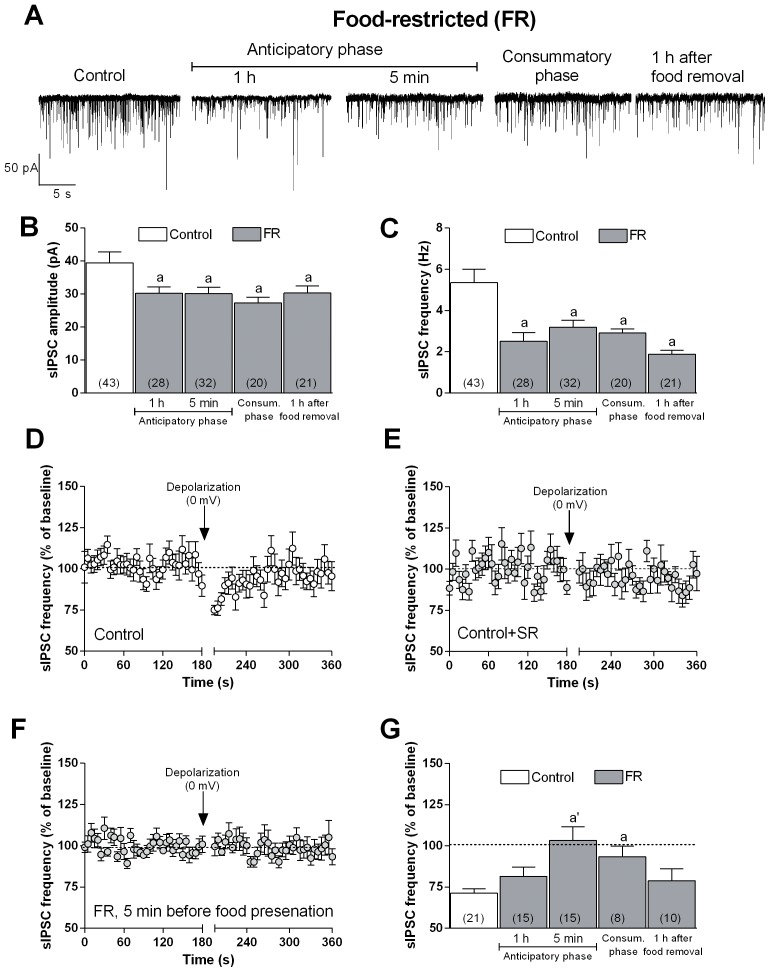
Changes in basal GABAergic sIPSCs and endocannabinoid-dependent DSI in mPFC principal neurons of FR rats during anticipatory and consummatory phase. (**A**) Representative traces of sIPSCs recorded from voltage-clamped (–65 mV) mPFC neurons in brain slices from rats fed ad libitum as well as FR rats at various time points relative to food presentation. (**B, C**) Summary of sIPSC amplitude and frequency, respectively, measured for the indicated numbers of cells from rats fed ad libitum and FR rats as in (**A**). (**D**) Depolarization (5 s)–induced reduction in sIPSC frequency (DSI) in mPFC neurons from rats fed ad libitum (n = 21 neurons). (**E**) Lack of effect of the DSI protocol on sIPSC frequency in the presence of the CB1R antagonist SR141716 (1 µM) in mPFC neurons of rats fed ad libitum (n = 13 neurons). (**F**) DSI recorded in mPFC neurons from FR rats at 5 min before food presentation (n = 15 neurons). (**G**) Summary of the percentage change in sIPSC frequency from baseline induced by depolarization in mPFC neurons from rats fed ad libitum or FR rats at different times relative to food presentation. For all experiments data are means ± SEM, and two-way ANOVA and Bonferroni post-hoc test were performed, ^a^ *P*<0.05; ^a’^ *P*<0.0001 versus rats fed ad libitum.

The activity of GABAergic interneurons with their inhibitory synapses can regulate the excitability of mPFC glutamatergic neurons that project onto VTA and that, in turn, control the excitability of mesocortical dopaminergic neurons. Given that CB1 receptors are located in these inhibitory synapses [33] and that FR can alter the function of the eCB system [7], we next analyzed the basal properties of GABAA receptor–mediated sIPSCs recorded in voltage-clamped (–65 mV) pyramidal neurons in slices of the mPFC obtained from FR and fed ad libitum rats. Both the frequency and amplitude of sIPSCs were significantly reduced at 60 and 5 min before food presentation, during the consummatory phase (at the end of the 2-h period of food availability), as well as at 1 h after food removal in FR rats compared with values measured in neurons from rats fed ad libitum that had free access to food at all time. Two-way ANOVA revealed a significant main effect of treatment (fed ad libitum vs FR) [amplitude, F(1, 265) = 18.49; *P*<0.0001; frequency, F(1, 265) = 38.54, *P*<0.0001] (Fig. 5A–C). To determine whether the reduced sIPSC frequency detected in FR rats was attributable to a change in CB1 receptor function as well as CB1-mediated regulation of presynaptic GABA release, we studied depolarization-induced suppression of inhibition (DSI) that involves the release of eCBs in response to an increase in intracellular Ca^2+^ concentration triggered by postsynaptic depolarization. The eCBs released, acting via a retrograde mechanism, activate the presynaptic CB1 receptors, inducing a transient suppression of GABA release from presynaptic terminals. As expected from previous observations [19], [34], administration of a brief depolarizing pulse (from –65 to 0 mV for 5 s) to pyramidal neurons from rats fed ad libitum resulted in a transient and significant reduction in sIPSC frequency (Fig. 5D), with no changes in current amplitude (data not shown), an effect likely dependent by a decreased probability of GABA release from presynaptic terminals. This scenario was supported by the observation that the DSI was blocked by bath application of SR141716 (Fig. 5E). DSI was not appreciable in brain slices isolated from FR rats sacrificed during the anticipatory phase (5 min before food presentation)(Fig. 5F) or during the consummatory phase, but was again detectable 1 h after food removal. Two-way ANOVA revealed a significant main effect of treatment (fed ad libitum vs FR) [F(1,131) = 29.82; *P*<0.0001]; a significant main effect of time [F(3,131) = 3.27; *P*<0.05], and a significant interaction between factors [F(3,131) = 3.27; *P*<0.05] (Fig. 4G).

**Figure 6:**
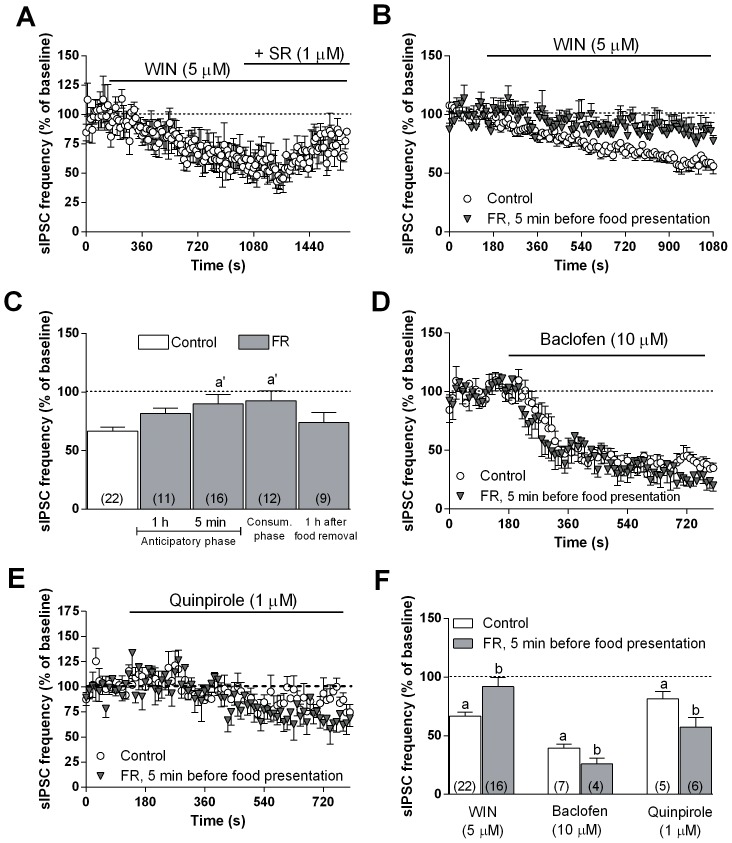
Changes in suppression of sIPSC frequency induced by WIN 55212-2, baclofen, or quinpirole in mPFC neurons of FR rats during anticipatory and consummatory phase. (**A**) Effect of 5 µM WIN 55212-2 and subsequent co-application of 1 µM SR141716 on sIPSC frequency in mPFC neurons from animals fed ad libitum (n = 6 neurons). (**B**) Effect of 5 µM WIN 55212-2 on sIPSC frequency in mPFC neurons from control and FR rats (sacrificed at 5 min before food presentation) (n = 16 to 22 neurons). (**C**) Summary of the percentage change in sIPSC frequency from baseline induced by WIN 55212-2 in mPFC neurons from control and FR rats (tested at different times relative to food presentation). (**D, E**) Effects of 10 µM baclofen and 1 µM quinpirole, respectively, on sIPSC frequency in mPFC neurons from control and FR rats (sacrificed at 5 min before food presentation) (n = 4 to 7 neurons). (**F**) Summary of the percentage change in sIPSC frequency from baseline induced by WIN 55212-2, baclofen, or quinpirole in control rats and FR rats (5 min before food presentation). Data are means ± SEM. Two-way ANOVA and Bonferroni post-hoc test, ^a^ *P*<0.05 versus baseline, ^a’^ *P*<0.01 versus control, ^b^ *P*<0.05 versus corresponding control.

To further evaluate the change in CB1 receptor-mediated regulation of presynaptic GABA release observed in FR rats, we also tested the effect of WIN 55212-2. As reported by previous reports [33], in mPFC slices from animals fed ad libitum, 10 min bath perfusion with 5 µM WIN 55,212-2 resulted in a marked decrease in sIPSC frequency, an effect that was antagonized by the subsequent co-application of 1 µM SR141716 (Fig. 6A). The WIN 55,212-2-induced effect was greatly attenuated in mPFC neurons from FR rats sacrificed 5 min before food presentation (Fig. 6B) or during the 2-h period of food consumption, but it returned to control values 1 h after food removal (Fig. 6C). Two-way ANOVA revealed a significant main effect of treatment (fed ad libitum vs FR) [F(1,135) = 21.84; *P*<0.0001] (Fig. 6C).

The observation that the apparent sensitivity of CB1 receptors to endogenous and exogenous agonist stimulation was reduced in FR rats would be consistent with a reduced inhibitory control of presynaptic GABA release and with a consequent increase in the probability of neurotransmitter exocytosis. However, this was not the case, as the basal frequency of sIPSCs in FR rats was reduced compared with that in rats fed ad libitum (Fig. 5A–C). To investigate this discrepancy and to better understand whether a presynaptic additional mechanism might contribute to the modulation of GABA release, we examined a possible role for GABAB and D_2_ dopamine receptors, both involved in the control of GABA release and thought to be localized at the same GABAergic synapses at the mPFC [33]. To evaluate the possible changes in the function of these presynaptic receptors, we recorded in glutamatergic mPFC neurons the sIPSCs in the presence of baclofen and quinpirole, agonists of GABAB and D_2_ receptors, respectively. Bath application of baclofen (10 µM) or quinpirole (1 µM) induced a significant decrease in sIPSC frequency in mPFC slices from rats fed ad libitum, but the effect of each agonist was significantly (T-test, *P*<0.05) more pronounced in the FR animals sacrificed 5 min before food consumption, the time point in which we observed the marked alteration in CB1 receptor-mediated modulation of sIPSC frequency (Fig. 6D–F).

### Changes in Neuronal Excitability in mPFC and VTA Projecting Neurons in Rats Subjected to FR

**Figure 7:**
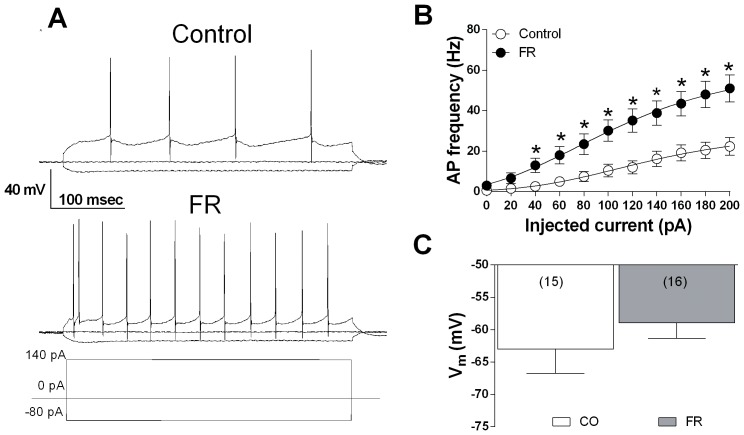
Changes in mPFC pyramidal neuron excitability in FR rats. (**A**) Representative membrane voltage responses to 400 msec of negative (−80 pA) and positive (140 pA) current pulses applied to single mPFC pyramidal neurons of control and FD rats (sacrifice 5 min before food consumption). (**B**) Scatter plot representing the quantitative effect of increasing depolarizing current steps on APs frequency in mPFC neurons of rats fed ad libitum and FR animals. ^a^ *P*<0.0001, two-way ANOVA. Data are expressed as means ± SEM and were obtained from 8 animals, and included 15 cells from rats fed ad libitum, and 16 cells from FR animals. (**C**) Bar graph representing the resting membrane potential of mPFC principal neurons, for the indicated numbers of cells, from fed ad libitum and FR rats.

The reorganization of presynaptic receptors involved in the control of inhibitory transmission onto mPFC GABAergic synapses could result, in turn, in an alterated excitability of the whole mesocortical circuitry. In order to test this hypothesis, we performed current-clamp recordings in single mPFC glutamatergic and VTA dopaminergic neurons. As shown in Fig. 7, in mPFC glutamatergic projecting neurons of FR rats sacrificed 5 min before food consumption, injection of depolarizing current steps (from 0 to 200 pA) evoked firing of action potentials (APs) whose frequency was markedly increased [F(1, 435) = 4.83, *P*<0.0001] compared to animals fed ad libitum (Fig. 7A, B). The resting membrane potential of cells from the two groups of animals was not significantly different (fed ad libitum: −63±3.7 mV; FR: −59±2.3 mV) (Fig. 7C).

**Figure 8:**
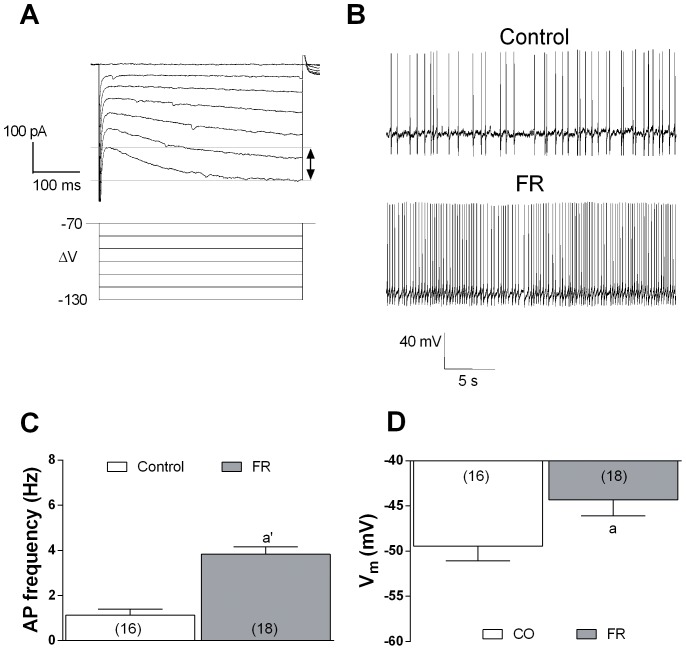
Changes in VTA dopaminergic neurons excitability in FR rats. (**A**) Dopaminergic neurons show a typical I_h_ current when the membrane is hyperpolarized starting from −100 mV. (**B**) Representative traces of spontaneous AP firing recorded from single VTA dopaminergic neuron from fed ad libitum and FR rats (sacrificed 5 min before food consumption). (**C**) Bar graph representing the quantitative effect of FR on spontaneous AP firing recorded in VTA dopaminergic neurons. ^a^ *P*<0.0001 vs rats fed ad libitum, unpaired t-Test. Data are calculated from 8 animals, including 16 cells from rats fed ad libitum, and 18 cells from FR animals. (**D**) Bar graph representing the resting membrane potential of VTA dopaminergic neurons, for the indicated numbers of cells, from fed ad libitum and FR rats. ^a^ *P*<0.05 versus fed ad libitum, unpaired t-Test.

Due to the their well established neurophysiological properties [35], VTA dopaminergic projecting neurons were easily identified on the basis of the presence of a typical *I*_h_ current sag that could be evoked in response to hyperpolarizing potentials (Fig. 8A). In agreement with their pace-maker properties [36], these neurons show a spontaneous firing of APs (Fig. 8B). The frequency of spontaneous APs resulted significantly increased (T-test, *P*<0.0001) in neurons of FR rats (3.8±0.32 Hz), tested 5 min before food consumption, compared to what observed in animals fed ad libitum (1.1±0.25 Hz) (Fig. 8B, C). The resting membrane potential of VTA dopamine neurons from FR rats (−44.3±1.6 mV) resulted significantly (T-test, *P*<0.05) lower than that found in rats fed ad libitum (−49±1.8 mV) (Fig. 6D).

### Antagonism of Acute SR141716 Treatment on FR-induced Effects

**Figure 9:**
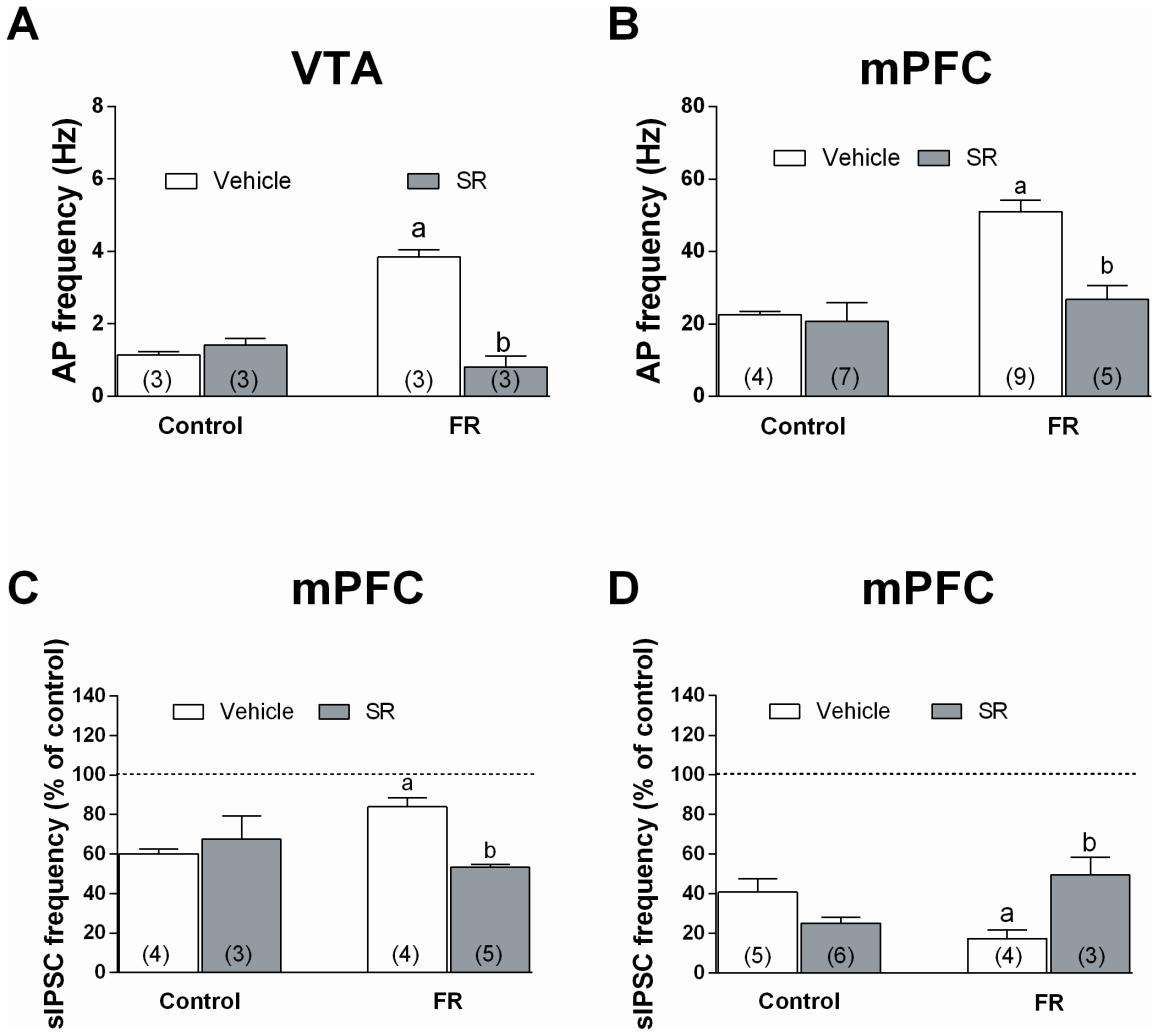
Effects of the systemic administration of SR141716 in fed ad libitum and FR rats on mPFC and VTA neurons excitability and inhibitory activity of WIN 55212-2 and baclofen on sIPSC frequency in mPFC neurons. SR141716 (1 mg/kg, i.p.) was administered 1 h before sacrifice; for FR rats, sacrifice corresponded to the 5 min preceding food consumption. (**A**) Bar graph representing the effect of the administration of SR141716 on spontaneous AP firing recorded in VTA dopaminergic neurons. Data are expressed as absolute values (Hz) and were obtained from 3 different cells for each experimental group. (**B**) Bar graph representing the effect of SR141716 on the frequency of depolarization-induced APs in mPFC neurons. Data are expressed as absolute values (Hz) and were obtained from 4 to 9 different cells for each experimental groups. (**C, D**) Bar graphs representing the effect of SR141716 on the inhibitory action of (**C**) WIN 55212-2 (5 µM) and (**D**) baclofen (10 µM) on sIPSC frequency in mPFC neurons. Data are expressed as percentage of control and were calculated from 4 to 6 different cells. ^a^ *P*<0.0001 versus fed ad libitum rats treated with vehicle; ^b^ *P*<0.0001 versus FR treated with vehicle. For all experiments one way ANOVA and Newman Keuls post hoc test were performed.

In order to understand whether the FR-induced changes observed in both mPFC and VTA principal neurons were consequent of CB1 receptor activation before food consumption, we treated a subset of animals with a single intraperitoneal injection of SR141716 (1 mg/kg, i.p) 1 h before food presentation. Current-clamp experiment performed in both mPFC and VTA revealed that the increase in spontaneous firing rate of VTA dopaminergic neurons as well as the depolarization-induced AP discharge in mPFC glutamatergic neurons induced by FR were completely antagonized in FR rats injected with SR141716 (Fig. 9A, B) [one-way ANOVA and Newman-Keuls test: F(3,11) = 55.24, *P*<0.0001; one-way ANOVA and Newman-Keuls test: F(3,24) = 4.683, *P*<0.05]. The injection of SR141716 1 h before food presentation was also able to restore in FR rats the inhibitory efficacy of WIN 55,212-2 on sIPSCs frequency (Fig. 9C) [one-way ANOVA and Newman-Keuls test: F(3,15) = 8.1, *P*<0.05 vs FR injected with vehicle] as well as the increased efficacy observed with baclofen perfusion in slices (Fig. 9D) [one-way ANOVA and Newman-Keuls test: F(3,24) = 4.683, *P*<0.05 vs FR injected with vehicle].

### Alteration of CB1 Receptor Expression in the mPFC of Food Restricted Rats

**Figure 10:**
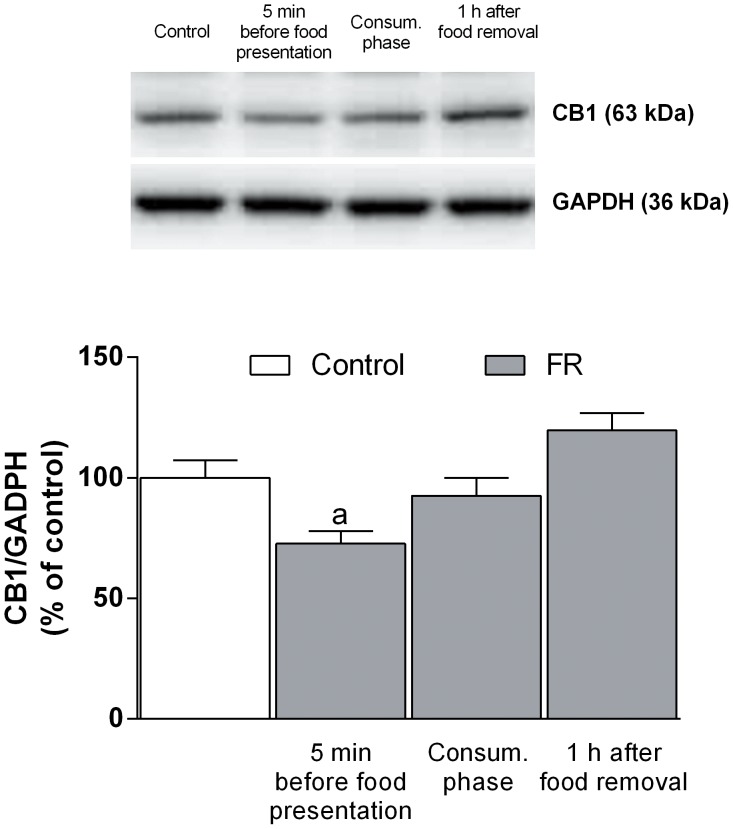
Effect of food restriction on CB1 receptor expression in the mPFC. Protein extracts prepared from the mPFC of fed ad libitum rats or FR rats at the indicated times relative to food presentation were subjected to immunoblot analysis with antibodies to CB1 and to GAPDH. A representative blot as well as quantitation of the CB1/GAPDH ratio (means ± SEM for 5 to 10 rats) are shown. ^a^ *P*<0.001 versus fed ad libitum rats, one way ANOVA with Bonferroni’s post hoc test.

As previously reported, expression of CB1 receptors protein has been found to be markedly affected by specific dietary schedules [9], [18], [10]. Accordingly, immunoblot analysis revealed that the amount of CB1 receptors was significantly decreased at 5 min before food presentation in FR rats [one-way ANOVA and Newman-Keuls test: *F*(6, 50) = 5.038, *P*<0.001] (Fig. 10). This effect was no longer apparent during the consummatory phase or after food removal.

**Figure 11:**
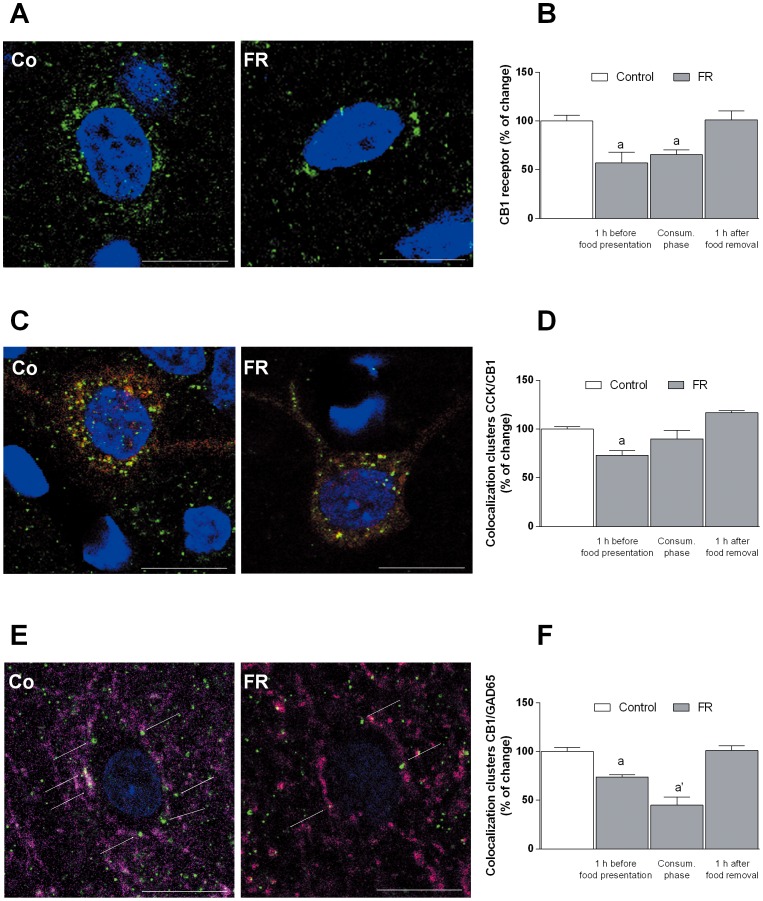
Effect of food restriction on the amount of CB1 receptors in mPFC. (**A**) Confocal images of CB1 receptor (green, nuclei blue); (**B**) Semiquantitative determination of the abundance of CB1 receptor, evaluated by image analysis of the immunohistochemistry, in mPFC of rat exposed to food restriction during anticipatory and consummatory phase with respect to fed ad libitum animals. (**C**) Confocal images of CB1 receptor (green) localization in CCK positive neurons (red, nuclei blue); (**D**) Semiquantitative determination of the abundance of the colocalization CCK/CB1 receptor, determined by image analysis of the immunohistochemistry data, in FR and fed ad libitum animals. (**E**) colocalization of GAD65/CB1 cluster (CB1 receptors green; GAD65 fuchsia, nuclei blue). (**F**) Semiquantitative determination of the abundance of the cluster GAD65/CB1 receptor as determined by image analysis of the immunohistochemistry data. Results are expressed as percentage of change in cluster numbers relative to fed ad libitum rats and are mean ± SEM of values of 5 rats for each experimental group. ^a^ *P*<0.005; ^a’^ *P*<0.001 versus fed ad libitum rats, one way ANOVA; image scale bar 10 µm.

As illustrated in Fig. 11 A–B, immunohistochemistry and confocal microscopy revealed that the amount of CB1 receptor immunostaining was significantly decreased (−43%) 1 h before food presentation, compared to rats fed ad libitum; such reduction was still observed during the consummatory phase (−35%), but was no longer apparent 1 h after food removal [one-way ANOVA and Scheffe’s test: F(3,16) = 8,020, *P* = 0.0017]. As previously reported [37]–[38], in the mPFC CB1 receptors are mainly expressed by CCK-containing GABAergic interneurons, whose axons target the perisomatic region of pyramidal neurons. We therefore analyzed the changes in CB1 receptor density induced by FR in CCK-positive neurons (Fig. 11C–D) as well as their co-localization with GAD65 at presynaptic GABAergic terminals (Fig. 11E–F). Double immunostaining for CB1 receptors and CCK resulted decreased (−27%) during the anticipatory phase (1 h before food presentation), did not change (−10%) during the consummatory phase, returning to control values 1 h after food removal (Fig. 11D) [one-way ANOVA and Scheffe’s test: F(3,16) = 11,963, *P* = 0.00023]. Principal neurons of the mPFC appear surrounded by puncta positive for both CB1 receptors and GAD65 immunostaining, confirming the localization of CB1 receptor on GABAergic terminals. Also co-localization density of CB1 receptors and GAD65 was significantly reduced, by 25 and 55%, during the anticipatory and consummatory phase, respectively, but not longer altered 1 h after food removal (Fig. 11F) [one-way ANOVA and Scheffe’s test: F(3,16) = 24,263, *P*<0.001].

## Discussion

In the present work we used a food restriction paradigm as an experimental model to emphasize the changes in dopamine output in the mPFC related to food presentation and the involvement of CB1 receptors in this complex mechanism. The observations that eating disorders are often associated with psychiatric diseases (such as depression, anxiety, and schizophrenia) and are related with an increased sensitivity to addictive drugs in both humans and rats [39], implicate both dopaminergic neurons and other neuromodulatory systems in their pathophysiology. We found that, in rats trained to consume their daily meal within a set 2-h period, the extracellular concentration of dopamine in the mPFC begins to increase 80 min before food presentation, rises further through the first 20 min of food intake, and then gradually returns to basal values by 40 min after food removal (Fig. 2). This biphasic increase in dopamine output (during food anticipation and consumption) is consistent with previous evidence suggesting that dopaminergic neurons in the mPFC are highly sensitive to the emotional state associated with reward prediction [40]. Our observation that rats fed ad libitum, that had free access to food all day, did not show any significant changes in basal dopamine extracellular concentration, further support this evidence.

In contrast, but still in agreement with previous data [6], [14], dopamine output in the shell of the NAcc showed only a small and transient increase after food presentation (Fig. 3). Moreover, stimulation of CB1 receptors by the acute administration of WIN 55,212-2 greatly enhanced the feeding-associated increase in dopamine output in the NAcc shell but had no such effect in the mPFC, consistent with the different roles of mesocortical versus mesolimbic dopaminergic neurons in response to salient stimuli (Fig. 2–3).

Our observation that both acute and chronic blockade of CB1 receptors with SR141716 markedly attenuated the increase in dopamine output in the mPFC during both the anticipatory and consummatory phases of food restriction, and that acute administration of WIN 55,212-2 enhanced dopamine output in the NAcc in response to food presentation- effect that was completely antagonized by blockade of CB1 receptors with SR141716- confirm a notable role for the eCB system in the regulation of dopaminergic neuron activity after food intake restriction. The fact that acute administration of WIN 55,212-2 failed to significantly modify the response of mPFC dopaminergic neurons to food restriction, is consistent with our present finding showing that CB1 abundance (Fig. 10) and function (Fig. 6) are down-regulated in the mPFC of FR rats and suggests that the FR-induced inhibition of CB1 receptor activity might be significantly correlated with the associated increase in dopamine output. In fact, evidences suggest that eCBs may modulate dopamine release in the PFC indirectly by activating CB1 receptors located on presynaptic GABAergic terminals, thereby inhibiting GABA release and resulting in a disinhibition of glutamatergic neurons that project to the VTA. An increase in glutamate release in the VTA would stimulate dopaminergic neurons that project back to the mPFC and thereby increase the release of dopamine in this region [20].

A role for CB1 receptors in the control of dopamine output in rats is well established [13]–[14], [20], [22]. As previously reported [20], GABAergic synapses in mPFC play an important role in the mesocortical feedback control of dopamine output. To further investigate the role of CB1 receptors and their regulatory control on mPFC GABAergic synapses in FR rats we examined the effect of the CB1 selective agonist WIN 55,212-2 and the DSI protocol onto mPFC inhibitory synapses to endogenous cannabinoids released in response to membrane depolarization in the DSI protocol. As expected [33], [41], both WIN 55,212-2 and depolarization-induced release of eCBs reduced sIPSC frequency in rats fed ad libitum through the activation of CB1 presynaptic receptors located in mPFC GABAergic terminals. This effect was not observed, however, during the anticipatory and consummatory phases in FR rats, suggestive of a down-regulation of CB1 receptors in these animals (Fig. 5, 6). Such down-regulation of CB1 receptors was supported by immunoblot (Fig. 10) and immunohistochemistry (Fig. 11) data revealing a significant decrease in the abundance of CB1 receptors in the mPFC of FR rats during the anticipatory phase. Moreover, immunohistochemistry data showed that the decrease of CB1 receptors abundance was predominant in CCK positive neurons in mPFC of FR animals during the anticipatory and consummatory phase. The decrease was particularly significant for GABAergic GAD65/CB1 receptor positive puncta surrounding the glutamatergic neurons in mPFC. Finally, all the observed changes returned to control values 1 h after food removal. These results are consistent with previous studies showing a decrease in CB1 receptor mRNA levels in the cingulate cortex as a result of food restriction or palatable food consumption in rats [18], [10]. Taken together, these data suggest that the expression and function of CB1 receptors are altered during both the anticipatory and consummatory phases in FR rats, and they further support the notion that changes in feeding behavior and body weight might alter the normal function of such neuromodulatory systems [15]–[17], [42].

To assess the impact of the observed changes in synaptic function, in a more detailed evaluation of the contribution of CB1 receptors to the regulation of GABAergic transmission in the mPFC of FR rats, we found that the basal frequency of sIPSCs, mediated by GABAA receptors in pyramidal neurons, was markedly reduced in these animals compared with rats fed ad libitum, suggestive of a reduced probability of GABA release from presynaptic terminals (Fig. 5). This alteration may lead to a disinhibition of glutamatergic neurons and a consequent increase in dopamine output in the mPFC through the stimulation of dopaminergic neurons that originate in the VTA.

It has been shown that also CB1 receptors in the VTA can regulate the activity of dopaminergic neurons projecting both to mPFC and NAcc [21]–[22]. Our observation that both the intraperitoneal administration of SR141716 and the local perfusion of the drug via inverse dialysis directly into the mPFC were able to markedly reduce the increase in dopamine output elicited by food anticipation and consumption, suggests that CB1 receptors located in this brain area, rather than those located in the VTA, are responsible for the observed increase in dopamine output (Fig. 9). It should be noted, however that not all neurons in the VTA project to the mPFC. Our results demonstrate that the most significant change in DA levels can been observed in the mPFC rather than NAcc suggesting that the synaptic connection between VTA and mPFC should be the most affected by FR protocol.

**Figure 12:**
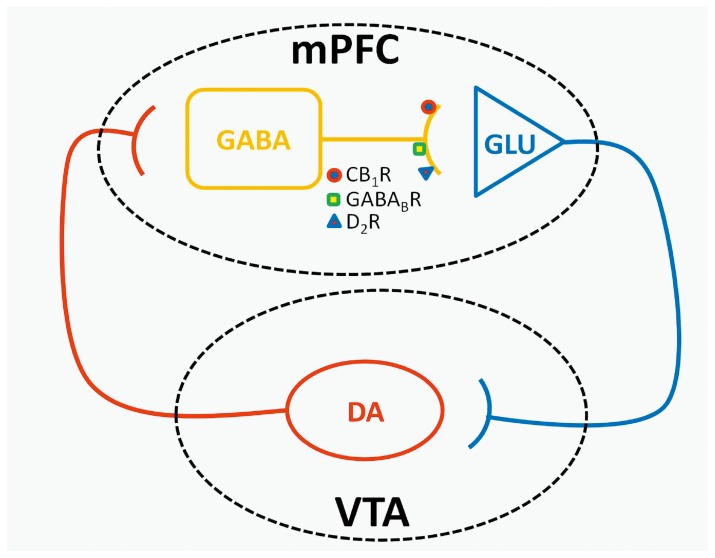
Representative scheme showing the main corticolimbic connections between the mPFC and VTA. The main dopaminergic pathways that come from the VTA to the mPFC is highlighted. The excitatory action of dopamine on GABAergic interneurons in the mPFC leads to an increase of GABA release that, in turn, inhibits the activity of glutamatergic projecting neurons that, from the mPFC, innervate again the VTA. The decrease in the glutamate tone in VTA contributes to the reduction of dopaminergic neurons firing with the consequent release of dopamine in the mPFC. This negative feedback control regulates the dopamine output in mPFC.

The reduced expression and function of CB1 receptors in the mPFC of FR rats, however, appeared inconsistent with the reduction in the frequency of basal GABAergic sIPSCs. Nonetheless, reduction of CB1 receptor function and expression may be crucial in triggering a possible adaptive mechanisms at a presynaptic level, involving other mechanisms that control the release of GABA. A presynaptic localization has been demonstrated for D_2_ dopamine receptors in the mPFC, as well as the colocalization of D_2_ and CB1 receptors at inhibitory synapses [43], [33]. D_2_ receptors, similar to CB1 and GABAB receptors [44]–[46], are coupled to G_i_ or G_o_ proteins and their activation inhibits presynaptic GABA release as a result of inhibition of protein kinase A and calcium influx in several brain areas including the mPFC [43], [47]–[50]. We have now shown a moderate but significant increase in the ability of quinpirole (D_2_ receptor agonist) or baclofen (GABAB receptor agonist) to reduce sIPSC frequency in FR rats, suggesting that up-regulation of both of these receptors may compensate for the reduced expression and function of CB1 receptors. Such rearrangement of presynaptic receptors, directed toward a decrease of CB1 and increase of GABAB as well as D2 receptors, might explain the reduced GABAergic sIPSC frequency that we observed in FR animals (Fig. 6). This effect, together to the enhanced membrane excitability (Fig. 7,8) found both in mPFC glutamatergic and VTA dopaminergic neurons cells, is consistent with the higher levels of dopamine observed in the mPFC of FR rats (Fig. 12).

Finally, our results showing that the down-regulation of CB1 receptors found in FR rats is associated with an increase in exploratory activity during the anticipatory phase are in line with recent data [51] that demonstrated in CB1 knockout mice a moderate enhancement in locomotor activity, consistent with the idea of an activation of the mesocortical neuronal circuitry (Fig. 4). An eCBergic tone-dependent regulation of locomotor activity was also suggested based on the alterations induced by SR141716A per se. On the other hand, a decrease in open-field activity in CB1 knockout mice was also found [51]. This apparent discrepancy could be related at least in part to a biphasic effects of eCBs in relation to their varying endogenous levels.

Taken together, our data suggest that the pronounced dopaminergic response in the mPFC of FR rats, associated with food anticipation and presentation, may be due to a remodeling of presynaptic mechanisms that contribute to the regulation of neurotransmitter release at GABAergic synapses in this brain region. We suggest that these changes might be triggered, in FR rats, by a decrease in the density and function of CB1 receptors in response to an enhanced release of eCB following food anticipation and consumption observed by other authors [7], that modifies the balance between the receptors expressed on GABAergic terminals and, in turn, their role in modulating dopamine output. Accordingly, in rats fed ad libitum our results are in agreement with previous observation where stimulation of CB1 receptors leads to a decrease in GABA output in the mPFC [20], [52]–[53]. In FR rats, the observed decrease in CB1 receptors density and function, that would suggest a reduced inhibition of GABA release, is compensated by an increase in the effect of the associated D_2_ and GABAB receptors, thus leading to a diminished probability of GABA release and an increase in dopamine output.

From these findings it can be concluded that the eCB system might work as a trigger of synaptic remodeling at mPFC GABAergic terminals leadings to an exacerbated response in dopamine neurons observed in mPFC of FR animals during food anticipation and consumption.

Overall, our data are consistent with those of previous studies and underline the role of eCBs in the control of neurotransmitter systems such as dopaminergic transmission that contribute directly to incentive and reward mechanisms in rodents and likely in humans [18], [10]. In conclusion, repeated exposure to scheduled feeding in rats might represent a useful animal model to study the profound changes in eCB, GABAergic, and dopaminergic systems in specific brain regions such as the mPFC. These changes underscore the important role of CB1 receptor signaling in the control of dopamine response to sustained motivated feeding behavior. Our results may provide a basis for the development of strategies that target CB1 receptors for the treatment of psychiatric diseases associated with changes in the feeding behavior.
